# Machine Learning Approach to Predict Ventricular Fibrillation Based on QRS Complex Shape

**DOI:** 10.3389/fphys.2019.01193

**Published:** 2019-09-20

**Authors:** Getu Tadele Taye, Eun Bo Shim, Han-Jeong Hwang, Ki Moo Lim

**Affiliations:** ^1^Department of Medical IT Convergence Engineering, Kumoh National Institute of Technology, Gumi, South Korea; ^2^Department of Mechanical & Biomedical Engineering, Kangwon National University, Chuncheon, South Korea; ^3^Department of IT Convergence Engineering, Kumoh National Institute of Technology, Gumi, South Korea

**Keywords:** prediction accuracy, QRS complex shape, QRS complex singed area, R-peak amplitude, ventricular fibrillation, ventricular tachyarrhythmia, ventricular tachycardia

## Abstract

Early prediction of the occurrence of ventricular tachyarrhythmia (VTA) has a potential to save patients’ lives. VTA includes ventricular tachycardia (VT) and ventricular fibrillation (VF). Several studies have achieved promising performances in predicting VT and VF using traditional heart rate variability (HRV) features. However, as VTA is a life-threatening heart condition, its prediction performance requires further improvement. To improve the performance of predicting VF, we used the QRS complex shape features, and traditional HRV features were also used for comparison. We extracted features from 120-s-long HRV and electrocardiogram (ECG) signals (QRS complex signed area and R-peak amplitude) to predict the VF onset 30 s before its occurrence. Two artificial neural network (ANN) classifiers were trained and tested with two feature sets derived from HRV and the QRS complex shape based on a 10-fold cross-validation. The prediction accuracy estimated using 11 HRV features was 72%, while that estimated using four QRS complex shape features yielded a high prediction accuracy of 98.6%. The QRS complex shape could play a significant role in performance improvement of predicting the occurrence of VF. Thus, the results of our study can be considered by the researchers who are developing an application for an implantable cardiac defibrillator (ICD) when to begin ventricular defibrillation.

## Introduction

Ventricular tachyarrhythmia (VTA) causes a rapid heart rate and eventual death in the absence of immediate medical intervention (Lee et al., 2016). As the majority of sudden cardiac deaths (SCD) occur because of VTA (Lee et al., 2016), early prediction of VTA is important to save patients’ lives. VTA contains different types of arrhythmias, such as ventricular tachycardia (VT) and ventricular fibrillation (VF). Because measuring and analyzing electrocardiogram (ECG) signals is an efficient way to identify electrical conduction malfunctions in the heart, such as arrhythmias, previous studies have attempted to predict the occurrence of VT, VF, or both by investigating electrocardiography (ECG) (Riasi and Mohebbi, 2013; Cappiello et al., 2015; Melillo et al., 2015).

Various methods have been introduced to predict VTA (VF, VT, or both), such as by assessing QRS (Q, R, and S waves in ECG) duration, T wave alternans, left ventricular impairment, QT (from the start of Q wave to the end of T wave in ECG) dispersion, and heart rate variability (HRV) (Lane et al., 2005). Among these, HRV is the most commonly employed signal that provides features for isolating arrhythmia from the normal HRV (Reed et al., 2005). HRV is a measure that indicates time variation in consecutive heartbeats, it is also denoted as RR (Billman et al., 2015). HRV has been analyzed to quantify its features using three analysis methods: time domain, frequency domain, and Poincare non-linear analyses (Bilgin et al., 2009; Joo et al., 2012; Lee et al., 2016).

Previous studies mainly used the above-mentioned three analysis methods to predict VT, VF, or both using HRV. Bilgin et al. (2009) performed a sub-band frequency analysis on HRV data and presented its feasibility on VTA prediction as compared to the traditional frequency analysis using two base-bands; low frequency (0.04–0.15 Hz) and high frequency (0.15–0.4 Hz). To obtain the sub-bands, they used a wavelet packet transform (WPT) and evaluated the sub-bands using a multilayer perceptron (MLP) neural network. Elias et al. used various features extracted in time and frequency domain (Ebrahimzadeh and Pooyan, 2011) to detect SCD early in patients with sustained VTA. They used an MLP neural network and k-nearest neighbors (KNN) to classify the healthy subjects and those prone to SCD, and principal component analysis (PCA) to reduce the feature dimensions. Recently, Elias et al. used both time-frequency and Poincare non-linear analyses to extract HRV features (Ebrahimzadeh et al., 2014). To evaluate the performance of their methods in the prediction of SCD in patients with sustained VTA, the features were extracted from different segments of HRV signals at successive 1-min intervals (i.e., the first, second, third, and fourth minute before the event). MLP and KNN were again used to classify healthy and VTA (Ebrahimzadeh et al., 2014).

Joo et al. (2012) predicted VT and VF 10 s before their occurrences using HRV features. They applied an artificial neural network (ANN) to deal with the complexities of the features extracted from the HRV (Joo et al., 2012). Recently, Lee et al. (2016) used the respiratory rate variability (RRV) features to improve the accuracy of VTA predictions using ANN. The performance of their predictions using RRV features outperformed HRV features.

Several studies showed promising performances of predicting VTA using HRV, RRV, other features, and their combination (Lee et al., 2016). However, they did not consider QRS complex features. The QRS complex represents the electrical activation of ventricles (Zhang et al., 1997) from which important features can be extracted. If more emphasis is placed on feature extraction from QRS complexes, performance of predicting VTA could be improved significantly.

The shape of the QRS complex provides abundant information about the pattern of the electrical propagation through ventricular tissue (Zhang et al., 1997). In clinical applications, the feature extraction and analysis of QRS complexes can predict ventricular arrhythmia, e.g., VF (Bassareo and Mercuro, 2013). Therefore, we hypothesize that the features from QRS complexes could be used to predict VTA in advance. The aim of this study was to investigate the feasibility of using the features extracted from QRS complexes for the early prediction of VTA (i.e., VF), as compared to traditional HRV features. To this end, we extracted two features such as QRS singed area and R-peak amplitude and investigated the prediction performance of VF using ANN, anticipating increased prediction performance using the features from the QRS complex. Also, four alternative machine learning algorithms showed a similar trend as ANN with high prediction performance using QRS shape features.

## Materials and Methods

### Dataset

We used the following freely available databases in PhysioNet (RRID:SCR_007345) (Goldberger et al., 2000): Creighton University (CU) ventricular tachyarrhythmia (CUDB) (Nolle et al., 1986), normal datasets from paroxysmal atrial fibrillation (PAF) prediction challenge database (PAFDB) (Moody et al., 2001) and the MIT-BIH normal sinus rhythm database (NSRDB) (Goldberger et al., 2000). The sampling frequencies were 250 Hz for the CUDB and 128 Hz the other two databases. Although there were 35 recordings in the CUDB, seven recordings were not considered because some contained only VT events (not VF events) and others were shorter than the required data length (>150 s). Thus, a total of 27 recordings were used for data analysis. The control group consisted of 28 subjects (22 subjects who did not have fibrillation events from the PAFDB and 6 subjects from the NSRDB).

### Preprocessing

**FIGURE 1:**
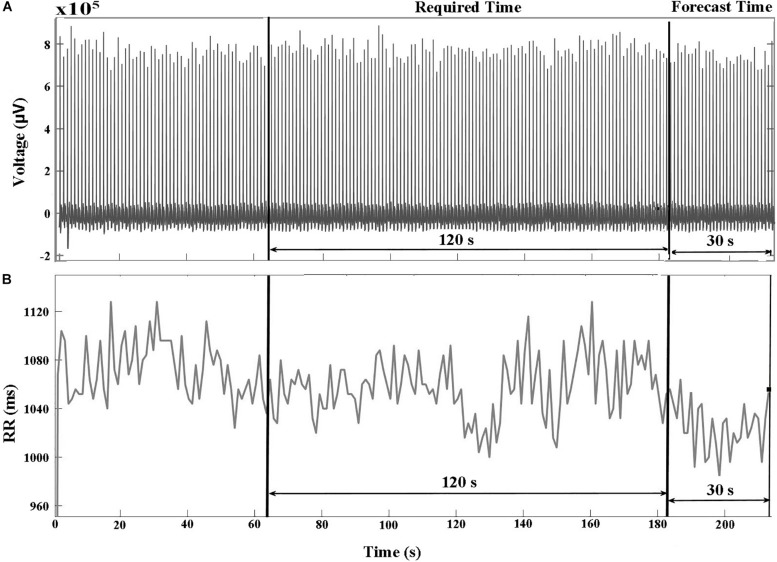
**(A)** ECG and **(B)** HRV signals of a VF subject that include two pre-VF regions. The signal 30 s before the VF onset is called the forecast time and the signal 120 s before the forecast time is called as required time. RR represents R-peak to R-peak interval in milliseconds (ms).

The databases in PhysioNet (PhysioNet, RRID:SCR_007345) (Goldberger et al., 2000) contained raw ECG signals and their corresponding HRV signals. The R-peak to R-peak intervals (RR) were produced by reading the annotation files in PhysioNet (PhysioNet, RRID:SCR_007345) (Goldberger et al., 2000) that were annotated by Cardiologists. Figures 1A,B show examples of ECG and HRV signals before VF occurred, respectively. We divided the signal into two parts: required and forecast time. The required time represents the time period used for feature extraction between 150 and 30 s before the VF onset time. The forecast time is the time period between 30 and 0 s before VF onset. Using the required time data, we could predict the occurrence of VF before the forecast time.

### Feature Extraction

Features were extracted from 27 VF and 28 control datasets. The descriptions of the features used in this study are listed in Table 1 (Lee et al., 2016). These features consist of 11 HRV features (4 features in the time domain, 4 features in the frequency domain, 3 features using Poincare non-linear analysis) and 4 QRS complex features (in the time domain). The desired features for the investigation were extracted from the required time between 150 and 30 s before VF onset. The QRS complex signed area and R-peak amplitude were computed using a function known as ECG-derived respiratory (EDR) from the PhysioNet (PhysioNet, RRID:SCR_007345) MATLAB toolbox (Moody et al., 1985). The method in EDR uses the effect of modulation of the R-peak amplitude, which is evaluated by processing the signed area under QRS complex in the ECG signal (Mazzanti et al., 2003). We computed the QRS complex signed area using the weighted sum of the samples between two boundaries: the interval from the PQ junction (the point where P wave and Q wave meet in ECG) to J-point (beginning of ST segment) (Moody et al., 1985). The R-peak amplitude was also computed by selecting a sample that had maximum value between the boundaries. The RR (in the equations) represents the R-peak to R-peak interval. In this end, the following 4 QRS features were used for early prediction of VF: mean and standard deviation of the QRS complex signed area and the R-peak amplitude.

**TABLE 1:** Features extracted from the HRV, the QRS complex singed area, and the R-peak amplitude.

**Component**	**Analysis**	**Feature**	**Unit**	**Description**
HRV	Time domain analysis	Mean NN	ms	Mean of normal R-peak to normal R-peak (NN) interval
		SDNN	ms	Standard deviation of NN intervals
		RMSSD	ms	Square root of the mean squared differences of successive NN intervals
		pNN50	%	Proportion of interval differences of successive NN intervals greater than 50 ms
	Frequency domain analysis	VLF	ms^2^	Power in very low frequency range (0–0.04 Hz)
		LF	ms^2^	Power in low frequency range (0.04–0.15 Hz)
		HF	ms^2^	Power in high frequency range (0.15–0.4 Hz)
		LF/HF		Ratio of LF over HF
	Poincare non-linear analysis	SD1	ms	Standard deviation of points perpendicular to the axis of line of identity
		SD2	ms	Standard deviation of points along the axis of line of identity,
		SD1/SD2		Ratio of SD1 over SD2
QRS complex signed are	Time domain analysis	QRSaM	μV	Mean of the QRS complex signed area
		QRSaSD	μV	Standard deviation of the QRS complex signed area
R-peak amplitude	Time domain analysis	RPampM	μV	Mean of the R-peak amplitude
		RPampSD	μV	Standard deviation of the R-peak amplitude

#### HRV Features

All HRV features were computed from successive RR intervals.

#### Time Domain Features

Four HRV features were computed in this category: (1) mean RR intervals [Mean NN (RR)], (2) standard deviation of NN (RR) intervals (SDNN), (3) square root of mean squared difference of successive NN (RR) intervals (RMSSD), and (4) the proportion of interval differences of successive NN (RR) intervals greater than 50 ms by the total number of NN (RR) intervals (pNN50), defined as follows:

(1) M⁢e⁢a⁢n⁢N⁢N=1/N⁢∑R⁢R⁢(i),

(2) $$SDNN=\sqrt{1/N\sum (RR\left(i+1\right)-MeanNN}){}^{2},$$

(3) $$RMSSD=\sqrt{1/N\sum (RR\left(i+1\right)-RR}\left(i\right)){}^{2},$$

(4) $$p\,N\,N\,50=\frac{\left|R\,R\,\left(i+1\right)-R\,R\,\left(i\right)\right|>50\,m\,s}{T\,o\,t\,a\,l\,n\,u\,m\,b\,e\,r\,o\,f\,R\,R\,i\,n\,t\,e\,r\,v\,a\,l\,s}\times 100.$$

##### Frequency domain features

We considered three frequency bands, such as the very low frequency (VLF) band (0–0.04 Hz), low frequency (LF) band (0.04–0.15 Hz), high frequency (HF) band (0.15–0.4 Hz), and the ratio of LF and HF. We computed the power spectrum density (PSD) of the bands using Welch’s periodogram with a Hanning window (window size: 256 points with an overlap of 50%).

##### Poincare non-linear features

The Poincare non-linear features were dispersion of points perpendicular and points along the axis of the line-of-identity. The standard deviation of the successive RR intervals scaled by 1/v2 (SD1) and the standard deviation of points along the axis of line-of-identity (SD2) were both calculated using (5) and (6). We considered the ratio of SD1 and SD2 as well.

(5) $$S\,D\,1=\sqrt{\frac{1}{2}\,V\,a\,r\,\left(R\,R\,\left(i\right)-R\,R\,\left(i+1\right)\right)},$$

(6) $$S\,D\,2=\sqrt{2\,S\,D\,N\,N^{2}-\frac{1}{2}\,S\,D\,1^{2}}.$$

#### QRS Complex Features

The QRS complex shape includes Q, R, and S waves from which the signed areas and the R-peak were calculated. The mean for QRS complex signed area and R-peak amplitude of the ECG were calculated using (7) and (8), and their standard deviations were calculated using (9) and (10).

(7) Q⁢R⁢S⁢a⁢M=1/N⁢∑|Q⁢R⁢S⁢s⁢i⁢g⁢n⁢e⁢d⁢a⁢r⁢e⁢a|,

(8) R⁢P⁢a⁢m⁢p⁢M=1/N⁢∑R⁢p⁢e⁢a⁢k,

(9) $$Q\,R\,S\,a\,S\,D=\sqrt{1/N\sum {\left(|QRSsignedarea|-QRSaM\right)}^{2},}$$

(10) $$R\,P\,a\,m\,p\,S\,D=\sqrt{1/N\sum {\left(Rpeak-RPampM\right)}^{2}.}$$

### Prediction Algorithms

**FIGURE 2:**
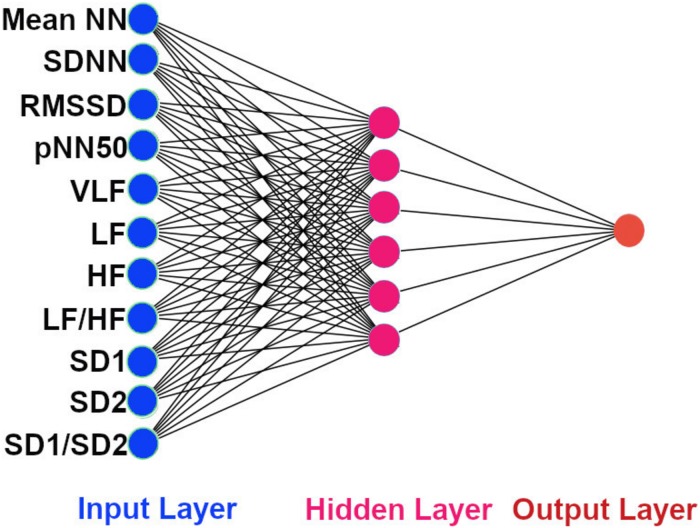
The architecture of our ANN using 11 HRV features. The input features to the ANN. Mean NN: mean normal R-peak to normal R-peak interval, SDNN: standard deviation of NN, RMSSD: square root of mean squared difference of successive NN, pNN50: proportion of interval differences of successive NN intervein greater than 50 ms, VLF: very low frequency, LF: low frequency, HF: high frequency, SD1: standard deviation of points perpendicular to the axis of line of identity, SD2: standard deviation of points along the axis of identity, and the ratio of SD1 and SD2.

**FIGURE 3:**
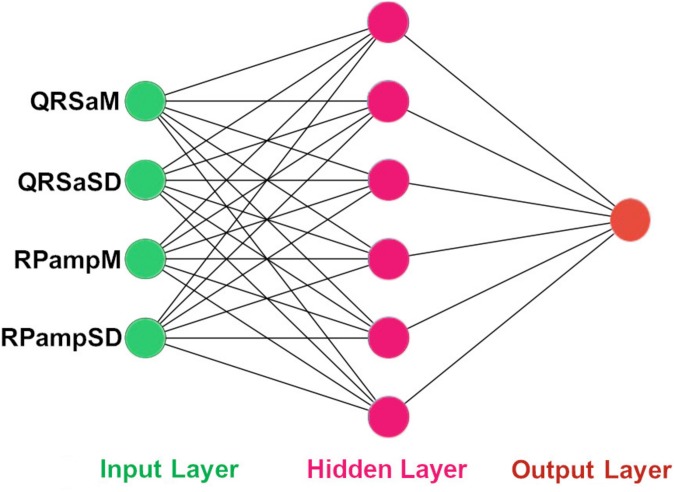
The architecture of our ANN using 4 QRS features. The input features to the ANN. QRSaM: mean of QRS complex signed areas, QRSaSD: standard deviation of QRS complex signed areas, RPampM: mean of R-peak amplitudes, and RPampSD: standard deviation of R-peak amplitudes.

**FIGURE 4:**
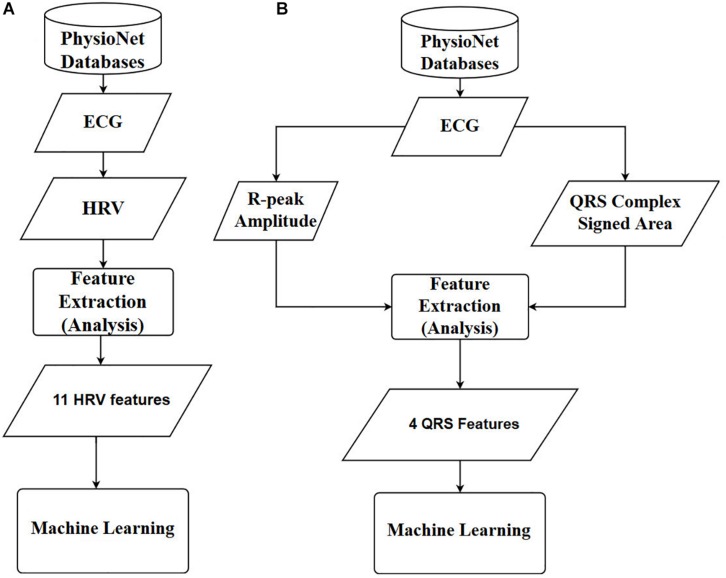
Overall diagrams of the proposed approach for early prediction of VF. **(A)** Using 11 HRV features. **(B)** Using 4 QRS complex features. ECG: electrocardiography, HRV: heart rate variability, ANN: artificial neural network.

The architecture of our ANN was a fully connected network structure consisting of three layers: an input layer with nodes representing input variables to the problem, a hidden layer containing nodes to help capture the non-linearity of the input data, and an output layer with a node representing the dependent variable (Figures 2, 3) (Lippmann, 1989; Basheer and Hajmeer, 2000). The hidden layer consisted of six neurons which were selected by trial and error (Figures 2, 3) with rectified linear unit (RELU) (Glorot et al., 2011) activation functions, and the output layer used a sigmoid activation function (Narayan, 1997). Activation functions decide which neurons should be activated or deactivated (Leshno et al., 1993). We implemented two ANN models with two different input parameters: 11 HRV features (Figure 2) and 4 QRS shape features (Figure 3).

We randomly shuffled the input features, and then used StandardScaler function from sklearn preprocessing library to standardize the input features. The features were standardized by removing the mean and scaling to unit variance. The standard score *Z* of a feature *x* is calculated as:

z=(x−μ)/s, where μ is the mean and *s* is the standard deviation of input features of all datasets.

Hence, the governing equations for RELU (12) and sigmoid (15) activation functions are provided as following:

(11) $$X_{j}=\sum_{i=1}^{n}w_{i\,j}\,x_{i}$$

(12) $$f\,\left(X_{j}\right)=\left\{ \begin{array}{cc} 0\; & \text{for}\,X_{j}\leq 0\\ X_{j}\; & \text{for}\,X_{j}\geq 0 \end{array}\right.$$

(13) $$y_{j}=f\,\left(X_{j}\right)+b_{j}$$

(14) $$X_{k}=\sum_{j=1}^{m}w_{j\,k}\,y_{j}$$

(15) $$f\,\left(X_{k}\right)=\frac{1}{1-e^{-X_{k}}}$$

(16) $$y_{k}=f\,\left(X_{k}\right)+b_{k}$$

where *x*_*i*_ is an input feature to the hidden layer, *w*_*ij*_ is a weight of the connection between *i*th input and *j*th neuron in the hidden layer, *X*_*j*_ is a weighted sum of the dot products of the input *x*_*j*_ and weight *w*_*ij*_, and *n* is number of input features. The output from *j*th neuron of the hidden layer is *y*_*j*_ (13), which is computed by applying RELU activation function to *X*_*j*_ and adding bias *b*_*j*_. Also, the output *y*_*k*_ of the output layer is computed by applying sigmoid activation function to *X*_*k*_, which is the weighted sum of the dot products of *y*_*j*_ and a weight of the connection between *j*th neuron in the hidden layer and *k*th neuron in the output layer *w*_*jk*_, and adding bias *b*_*k*_. The *m* is number of neurons in the hidden layer.

The ANN was trained using Adam optimizer algorithm which updates the weights (Kingma and Ba, 2014). Adam is an adaptive learning rate optimization algorithm that has been designed for training neural network. Because we implemented a binary classification model, we used binary cross-entropy as loss function to measure the divergence between two probabilities distribution.

Furthermore, we implemented four more machine learning algorithms such as: support vector machine (SVM), KNN, random forest (RF), and Gaussian Naive Bayes (NB) classifiers. These machine learning algorithms were implemented using sklearn library in python3.

All algorithms were evaluated 10 times with a 10-fold cross validation, to avoid overfitting. In the 10-fold cross validation (Weiss and Kulikowski, 1991), the dataset was randomly divided into approximately 10 groups. One group was treated as the testing dataset, and the remaining 9 groups were used for training. The cross-validation was repeated 10 times.

Finally, the prediction accuracies were estimated by calculating the means and standard deviations of each model. To observe the statistical differences between HRV and QRS shape accuracies, we performed two tailed *t*-test for each model. Also, to check the statistical differences among the accuracies for all the machine learning algorithms, we computed one-way repeated-measures ANOVA with Tukey *post hoc* analysis for multiple comparisons. The flowcharts of the methods used in this study are shown in Figure 4, where Figures 4A,B are based on 11 HRV and 4 QRS complex features, respectively.

## Results

Table 2 shows the comparison of the means and standard deviations of the HRV and QRS complex shape features between the control and VF dataset (see Supplementary Materials for more illustration). Nine of the 15 features, SDNN, RMSSD, pNN50, LF, SD1, SD2, QRSaSD, QRsampM, and QRSampSD, show statistically significant differences (two tailed *t*-test, *p* < 0.05).

**TABLE 2:** Comparison of HRV and QRS complex shape features between control and VF dataset (see Supplementary Material for more illustration).

**Features**	**VFs dataset**	**Control dataset**	***p*-value**
			
	**Mean ± SD**	**Mean ± SD**	
Mean NN (ms)	796.34 ± 366.84	756.81 ± 157.17	0.609
SDNN (ms)	150.54 ± 179.60	46.74 ± 24.74	0.0044
RMSSD (ms)	184.66 ± 219.11	49.44 ± 40.51	0.0027
pNN50 (%)	27.34 ± 29.64	7.08 ± 9.27	0.0013
VLF	17025.4 ± 72281.15	430.57 ± 497.05	0.24
LF	2386.03 ± 4842.65	422.51 ± 666.33	0.042
HF	7636.09 ± 26494.28	300.85 ± 690.3	0.16
LF/HF	1.7 ± 1.8	2.97 ± 4.82	0.203
SD1	131.13 ± 155.72	35.06 ± 28.74	0.0026
SD2	164.05 ± 203.797	53.48 ± 26.22	0.0072
SD1/SD2	0.826 ± 0.364	0.648 ± 0.381	0.087
QRSaM	2.27*E*+06 ± 9.02*E*+06	2.147*E*+05 ± 5.75*E*+05	0.243
QRSaSD	2.88*E*+06 ± 2.596*E*+06	9.689*E*+04 ± 9.309*E*+04	9.6E-07
QRSampM	5.356*E*+05 ± 2.307*E*+05	9.38*E*+04 ± 1.186*E*+05	5.63E-08
QRSampSD	1.516*E*+05 ± 8.626*E*+04	10778.166 ± 8717.264	6.78E-08

Table 3 summarizes the performance of two ANNs with different feature types; HRV vs. QRS. Eleven HRV features achieved 72% prediction accuracy. The sensitivity and specificity were 65.68% and 98.44%, respectively. When using 4 features extracted from the QRS complex singed area and the R-peak amplitude, the prediction performance improved dramatically. The accuracy, sensitivity, and specificity were 98.6, 98.4, and 99.04%, respectively. The result shows that the QRS complex shape features extracted from the ECG could have an impact in predicting VF before its occurrence in terms of its prediction performance.

**TABLE 3:** The results for the ANN in predicting VF 30 s before its occurrence.

**ANN with**	**Input**	**Sensitivity**	**Specificity**	**Accuracy)**	**AUC**
	**parameters**	**(%)**	**(%)**	**(%)**	
HRV	11	65.68	98.44	72 ± 18.2	0.71
QRS signed area + R-peak amplitude	4	98.4	99.04	98.6 ± 4.7	0.99

Table 4 presents the average computational times required for training and testing ANN using HRV and QRS complex shape features. The computational times needed for training and testing ANN using 11 HRV parameters were 1545 and 0.72 ms, respectively. Similarly, the computational time needed for training and testing ANN using 4 QRS complex shape parameters were 1505 and 0.7 ms, respectively. Although the number of input features are different, there was no significant difference between the two ANN models in terms of computational time. Note that the training time was estimated while an ANN model was constructed, and the testing time was estimated while one sample produced a prediction result. The training and testing times were computed for each cross-validation step, and they were averaged.

**TABLE 4:** Computational time needed for training and testing ANN using 11 HRV and 4 QRS shape features.

**ANN with**	**Input parameters**	**Average computational time (ms)**	
		
		**Fitting (training)**	**Prediction (testing)**
HRV	11	1545.04	0.72
QRS signed area + R-peak amplitude	4	1505.1	0.7
			

**FIGURE 5:**
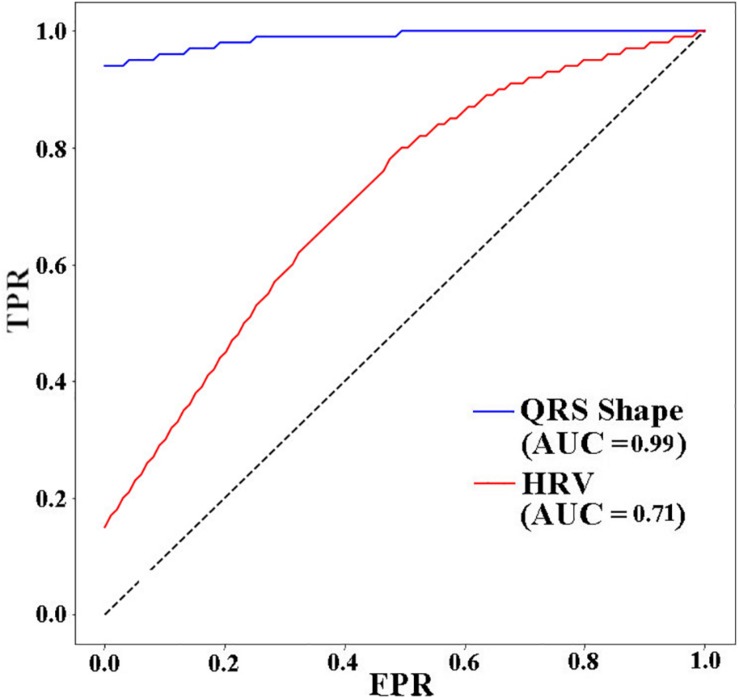
ROC AUCs (receiver operating characteristic area under curves) of ANNs for different input features used to predict VF 30 s before the occurrence. TPR: true positive rate, FPR: false positive rate.

Figure 5 presents the receiver operating characteristic (ROC) curve for the three models. The ANN with 11 HRV features has the lowest area under curve (AUC) value (0.71). When using 4 QRS complex shape features, the AUC reached 0.99.

**FIGURE 6:**
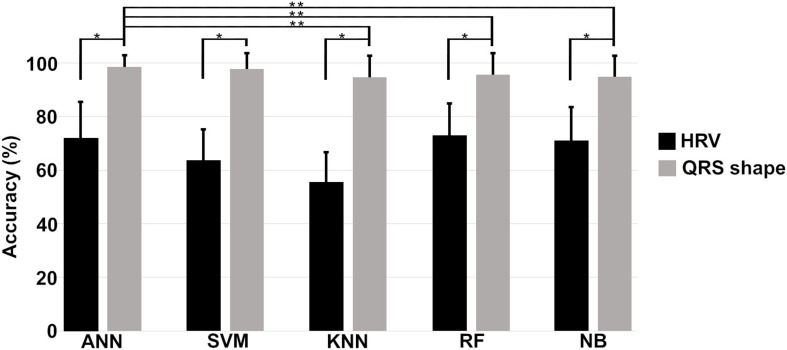
Means and standard deviations of the prediction accuracies of each algorithm. Single asterisk (^∗^) indicates a statistically significant difference between prediction accuracies using HRV and QRS shape features (QRS > HRV, *p* < 0.001), and double asterisk (^∗∗^) between the prediction accuracies of different algorithms (ANN = SVM > KNN, RF, and NB, *p* < 0.001). HRV: heart rate variability with 11 parameters, QRS shape: QRS signed area and R-peak amplitude with 4 parameters, ANN: artificial neural network, SVM: support vector machine, KNN: k-nearest neighbors, RF: random forest, NB: Gaussian Naïve Bayes.

Figure 6 shows the means and standard deviations of the accuracies evaluated 10 times using a 10-fold cross validation for all machine algorithms we considered. The performances of the QRS shape features statistically outperform those of the HRV features for all machine algorithms (two tailed *t*-test, *p* < 0.001). The accuracies of all algorithms showed statistically significant differences when using the QRS shape features [one-way ANOVA: *F*(4,485) = 4.43, *p* = 0.0016]. A *post hoc* test showed that the ANN exhibited a statistically higher performance than other algorithms (*p* < 0.05), except SVM (*p* = 0.95) (see Supplementary Material for more detailed results).

## Discussion

In this study, we showed that the performance for predicting VF can be improved by using features extracted from the QRS complex shape (QRS complex signed area and R-peak amplitude). A maximum prediction accuracy of 98.6% was obtained using ANN when only 4 features of the QRS complex signed area and R-peak amplitude were used. However, using HRV features presented a significantly low performance with a prediction accuracy of 72%. The ROC curve in Figure 5 also showed the same trend. From the result in Table 3, we demonstrated that QRS shape features can predict VF before its occurrence more accurately than the traditional HRV features.

As depicted in Figure 6, the prediction performance obtained using 4 QRS shape features was statistically higher than that was obtained using 11 HRV features for all algorithms. Also, ANN statistically outperformed three algorithms, such as KNN, RF, and NB, when using 4 QRS features, and its performance was comparable with SVM. However, ANN needed computational time more than the other algorithms to train the model, but not for testing time; all algorithms, including ANN, need less than 1 ms for one testing (Supplementary Table 2).

The QRS complex of an ECG contains information about the ventricular depolarization process. The time at which the QRS complex is generated is the time required to complete ventricular depolarization. The QRS amplitude is proportional to the energy consumed for ventricular depolarization. Therefore, if the electrical properties of the ventricular tissue remain unchanged, the QRS shape does not change within a short period of time (several seconds to several hours). How could we predict the VTA shortly before using QRS shape information? To our best knowledge, the reason is that when the ventricles begin to make reentrant waves due to ectopic focus or other reasons, the cardiac electrical wave pattern begins to change (which can affect the QRS shape). In addition, when electrical waves are different, the way in which the ventricles contract is also different, which changes the location of the ventricular tissue that is the source of the ECG. Changes in the location of ventricular tissue will affect the ECGs that reflect this. Therefore, QRS complex shape represents the depolarization (activation) of the ventricle muscles (Zhang et al., 1997) from which abnormalities in the electrical activation features can be extracted for early prediction of VF. The findings highlight the importance of the QRS complex shape features for predicting VF.

The means and standard deviations presented in Table 2 show the comparison between the features of VF and control datasets. Nine features have statistically significant differences between the VF and control groups (*p* < 0.05). We could use these features to compare the VF and control groups. However, the prediction of VF is not achievable by mere comparison of some parameters but by classification of complex patterns of every feature based on machine learning techniques.

Previous studies dealt with features extracted from HRV and predicted VF, VT, or both with promising performance. Elias et al. showed a performance with 99.73% accuracy for features extracted from HRV 1 min just before the occurrence of SCD early in patients with sustained VTA (Ebrahimzadeh et al., 2014). However, they did not consider the forecast time which is a time period before the occurrence of the VTA. Joo et al. (2012) predicted VT and VF 10 s before the events occurred. They showed 76.6% accuracy for predicting VT and 92.2% accuracy for predicting VF. Recently, Lee et al. used RRV and HRV features to predict VT 1 h before it occurred. They showed a prediction accuracy of 85.3%, which is 10% better than their result when they used only HRV features (73.5%). The performance of our ANN model based on the QRS shape and HRV features was slightly higher than those of Elias et al. and Joo et al.’s result. Furthermore, we considered 30-s-long forecast time. However, Lee et al. considered a longer forecast time (1 h). Even though they considered longer forecast times, our ANN model showed higher accuracy.

Results show that features extracted from HRV contain important information for predicting the occurrence of VF several minutes in advance. However, Lee et al. (2016) revealed that the performance using only HRV features can be improved by adding RRV features. We found that only the QRS complex shape or that combined with HRV can improve the performance of predicting VF. In our study, we used 2-min-long signal to predict VF 30 s before its occurrence. The signals we used for the analysis (required time) and the prediction time gap (forecast time) were short. However, our study showed that the features extracted from QRS complex morphology (shape) could have effects for predicting VF.

We compared the performance obtained using a combination of HRV and QRS shape features with that obtained using only QRS shape features, but little improvement in prediction accuracy (only ∼1%) was found for the combination features. This indicates that using QRS shape features solely would be an efficient way to predict VF. Therefore, we decided to not include the result for the combination features in our study.

Our algorithm could be installed in patients’ implantable cardiac defibrillator (ICD) for real-time VF prediction as an additional functionality to VF detection. Predicting the occurrence of VF hours in advance would be more useful, however, the datasets used for this paper limited to 120 s data window and predict VF 30 s before its occurrence. Au-Yeung et al. (2018) showed that a correct prediction could be made when the ventricular arrhythmia occurs nearer. Thus, our prediction accuracy of 98.6% was higher than that of Bayasi et al. (2016) who predicted the occurrence of VF 3 h prior to the onset with an accuracy of 86%, and Lee et al. (2016) who predicted the onset 1 h prior with an accuracy of 85.3%.

According to the Task Force of the European Society of Cardiology and the North American Society of Pacing and Electrophysiology, a recording of approximately 1 min is needed to calculate the HF component, and at least 2 min, to calculate the LF component (Task Force of the European Society of Cardiology the North American Society of Pacing Electrophysiology, 1996). However, the VLF calculated from short-term recordings (<5 min) is an uncertain measure (Task Force of the European Society of Cardiology the North American Society of Pacing Electrophysiology, 1996). The VF datasets in this study were very short in length, therefore, we had to use 120 s data window for extracting features 30 s before VF occurs.

The limitation of our study was the small dataset (55 recordings) and the short length of the signals before the VF occurred. To implement a study for clinical purposes, our ANN model must be trained using more datasets.

## Conclusion

In this study, we used an ANN to predict the VF using features extracted from 120 s HRV signals, the QRS complex signed area, and the R-peak amplitude 30 s before VF occurrence. The datasets were collected from the popular physiological archive PhysioNet. Although the datasets utilized in this study were relatively small, the performance of the ANN was better using QRS shape features than that of traditional HRV features. This was consistently observed in all machine learning algorithms implemented in this study, which demonstrates the feasibility of using QRS shape features to accurately predict VF onset. This work requires further investigation using a greater number of datasets to confirm the clinical feasibility of our proposed approach. Finally, the results of this study could be used to predict when an ICD will begin ventricular defibrillation.

## Data Availability

Publicly available datasets were analyzed in this study. This data can be found here: https://www.physionet.org/.

## Author Contributions

This manuscript is the intellectual product of the entire team. GT designed the study, wrote the simulation source code and the manuscript, performed the data analysis, and interpreted the results. KL, ES, and H-JH reviewed and revised the whole manuscript based on the simulation results. All authors read and approved the final manuscript.

## Conflict of Interest Statement

The authors declare that the research was conducted in the absence of any commercial or financial relationships that could be construed as a potential conflict of interest.
