# Supplementary material for: Machine Learning Approach to Predict Ventricular Fibrillation Based on QRS Complex Shape
Source: Front Physiol. 2019 Sep 20;10:1193. doi: 10.3389/fphys.2019.01193 (PMC6764170; doi:10.3389/fphys.2019.01193)
Supplement: Supplementary file 1 [file Table_1.docx]

Supplementary Material

Machine Learning Approach to Predict Ventricular Fibrillation Based on QRS Complex Shape

Getu Tadele Taye^1^, Eun Bo Shim^3^, Han-Jeong Hwang^1*^, Ki Moo Lim^2*^

*** Correspondence:** Ki Moo Lim (kmlim @kumoh.ac.kr) and Han-Jeong Hwang (h2j@kumoh.ac.kr)

# Supplementary Figures

The features that were used in this study are provided in the form of bar charts in this section (supplementary Figure 1 - 4). Datasets for the VF and Control datasets are indicated by red and blue line, respectively. In addition, means and standard deviations of both categories are provided in tabular form along with the p-value.

Receiver operating characteristic area under curves of support vector machine (SVM), k-nearest neighbors (kNN), random forest (RF), and Gaussian Naïve Bayes (NB) using QRS shape are provided below in Supplementary Figure 5.

**Time domain features for HRV**


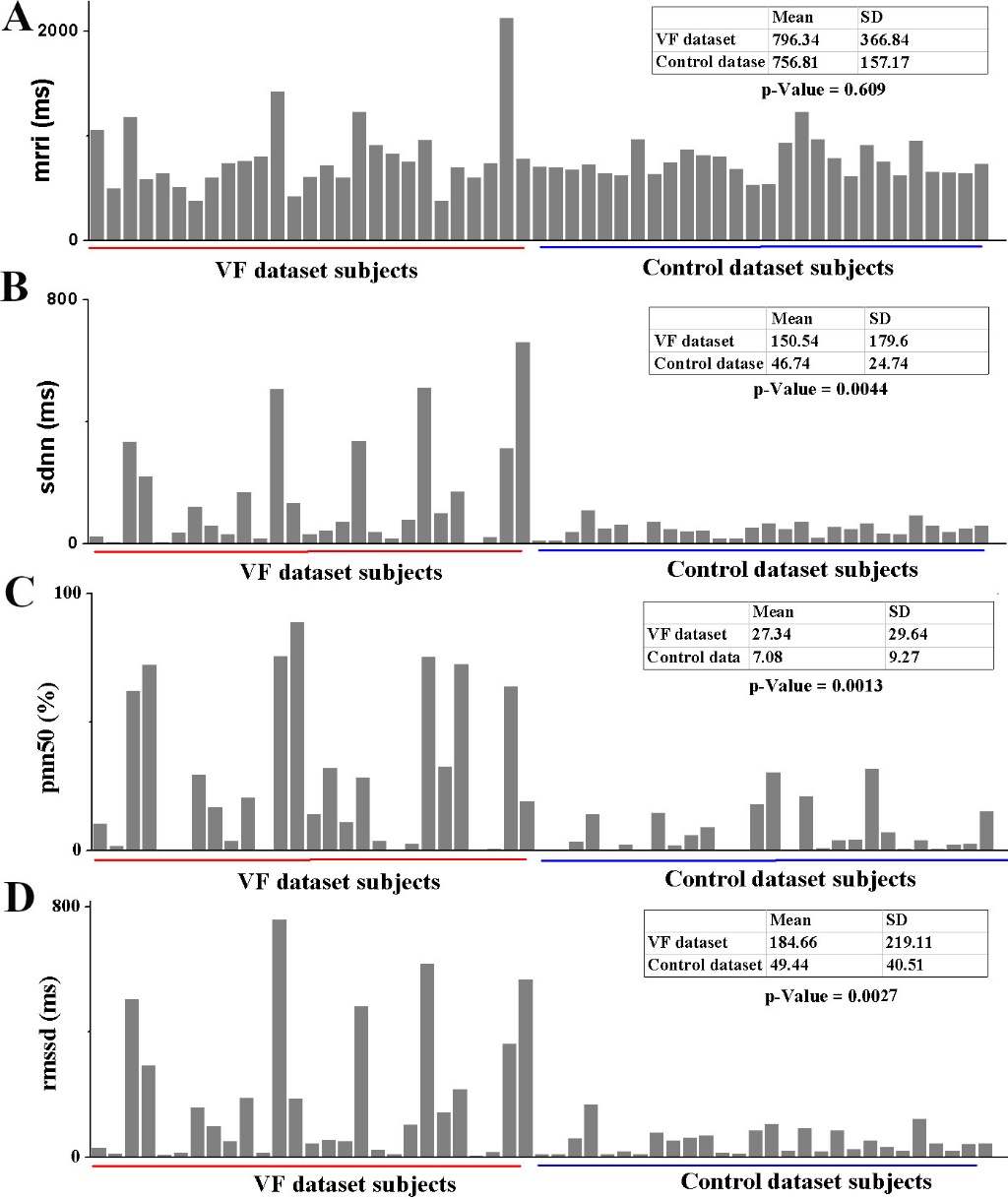


Supplementary Figure 1. (A) Mean of normal R-peak to normal R-peak (RR) interval (mrri). (B) Standard deviation of RR intervals (sdnn). (C) Proportion of interval differences of successive RR intervals greater than 50 ms (pnn50). (D) Square root of the mean squared differences of successive NN (RMSSD)

**Frequency domain features for HRV**

**
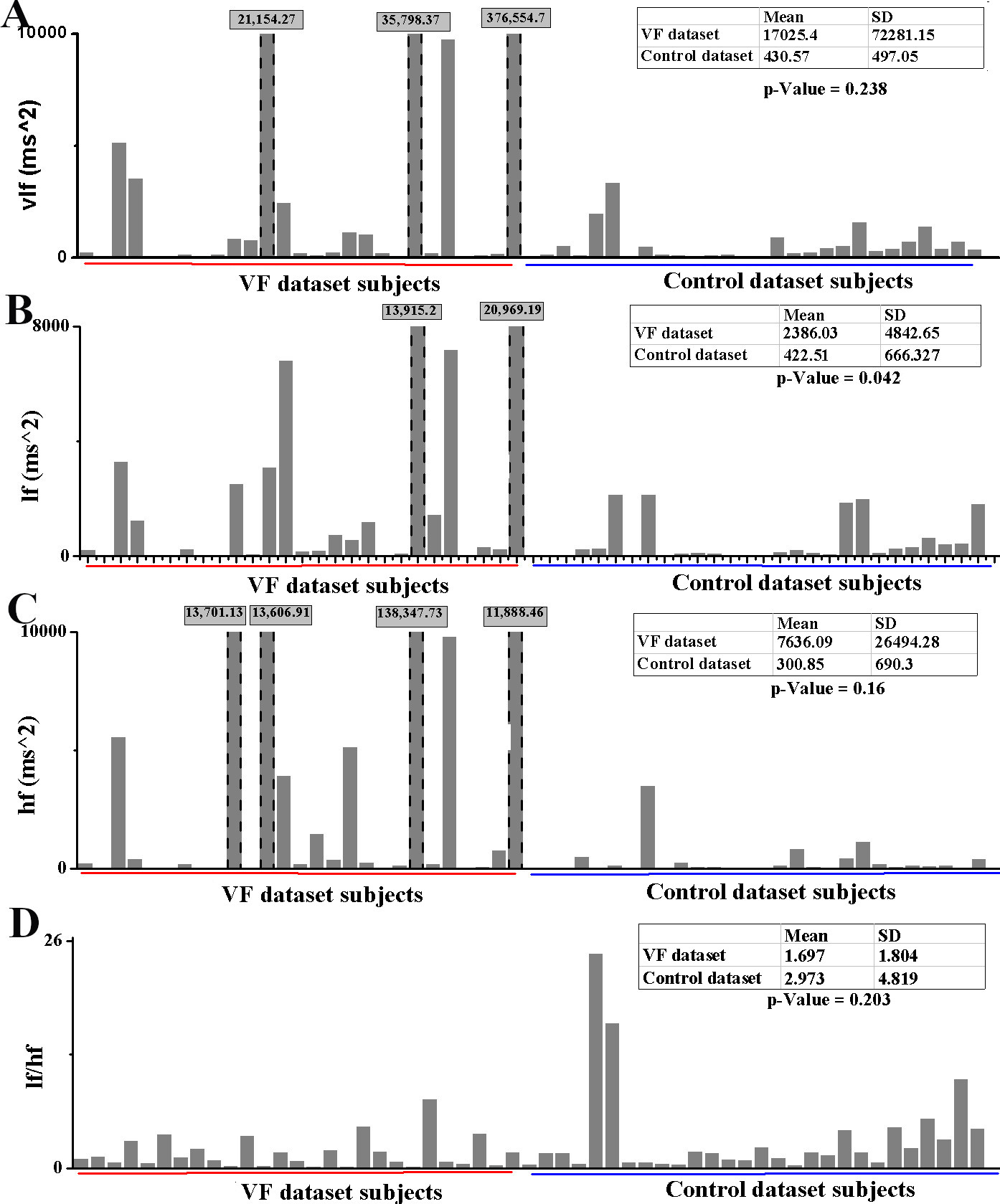
**

Supplementary Figure 2. (A) Power in very low frequency range (0–0.04 Hz) (VLF). (B) Power in low frequency range (0.04–0.15 Hz) (LF). (C) Power in high frequency range (0.15–0.4 Hz) (HF). (D) Ratio of LF to HF (LF/HF).

**Poincare nonlinear features for HRV**


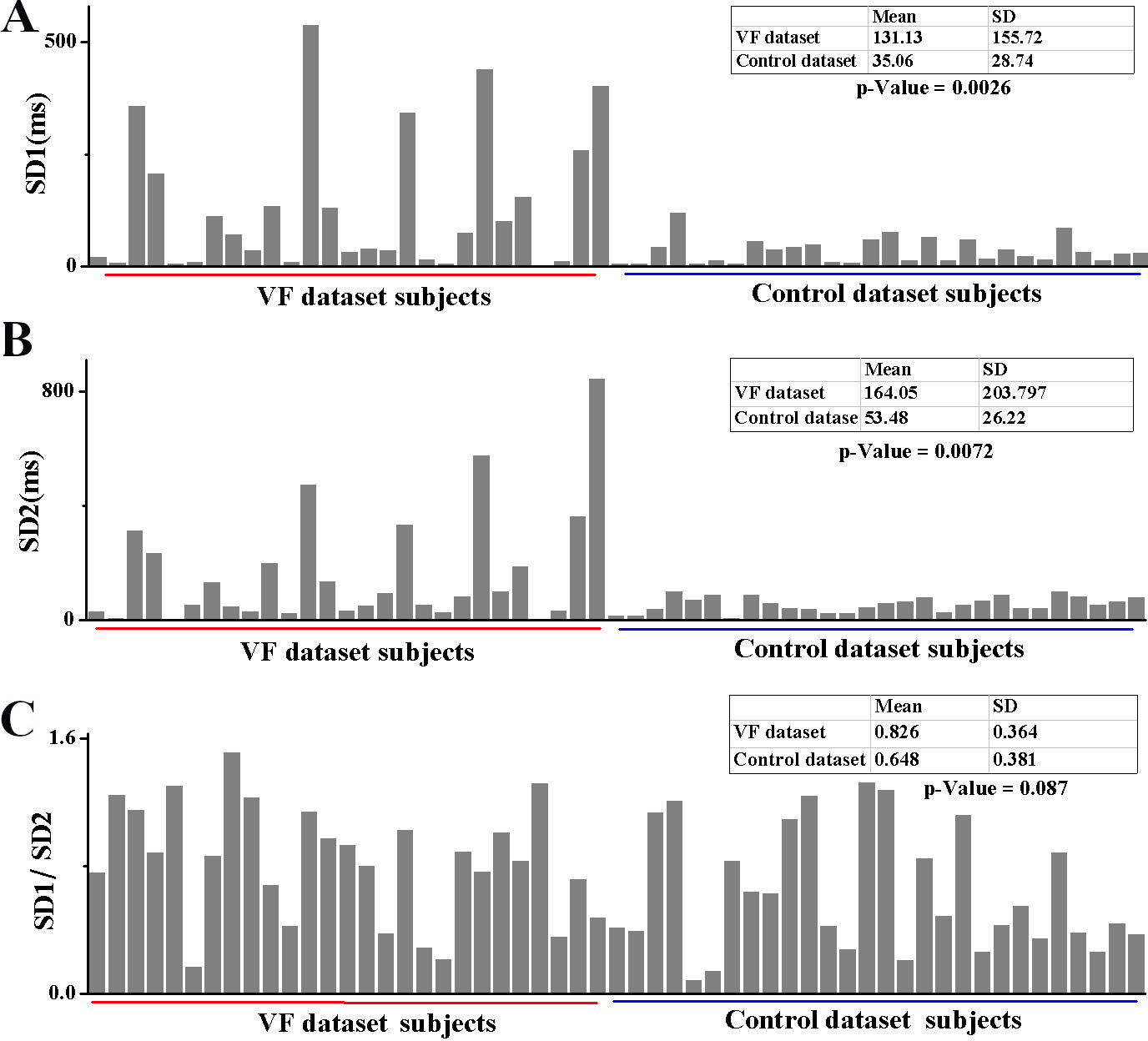


Supplementary Figure 3. (A) Standard deviation of points perpendicular to the axis of line of identity (SD1). (B) Standard deviation of points along the axis of line of identity (SD2). (C) Ratio of SD1 to SD2 (SD1/SD2).

**Time domain feature for QRS Complex Shape (QRS Complex signed area and R-peak amplitude)**

**
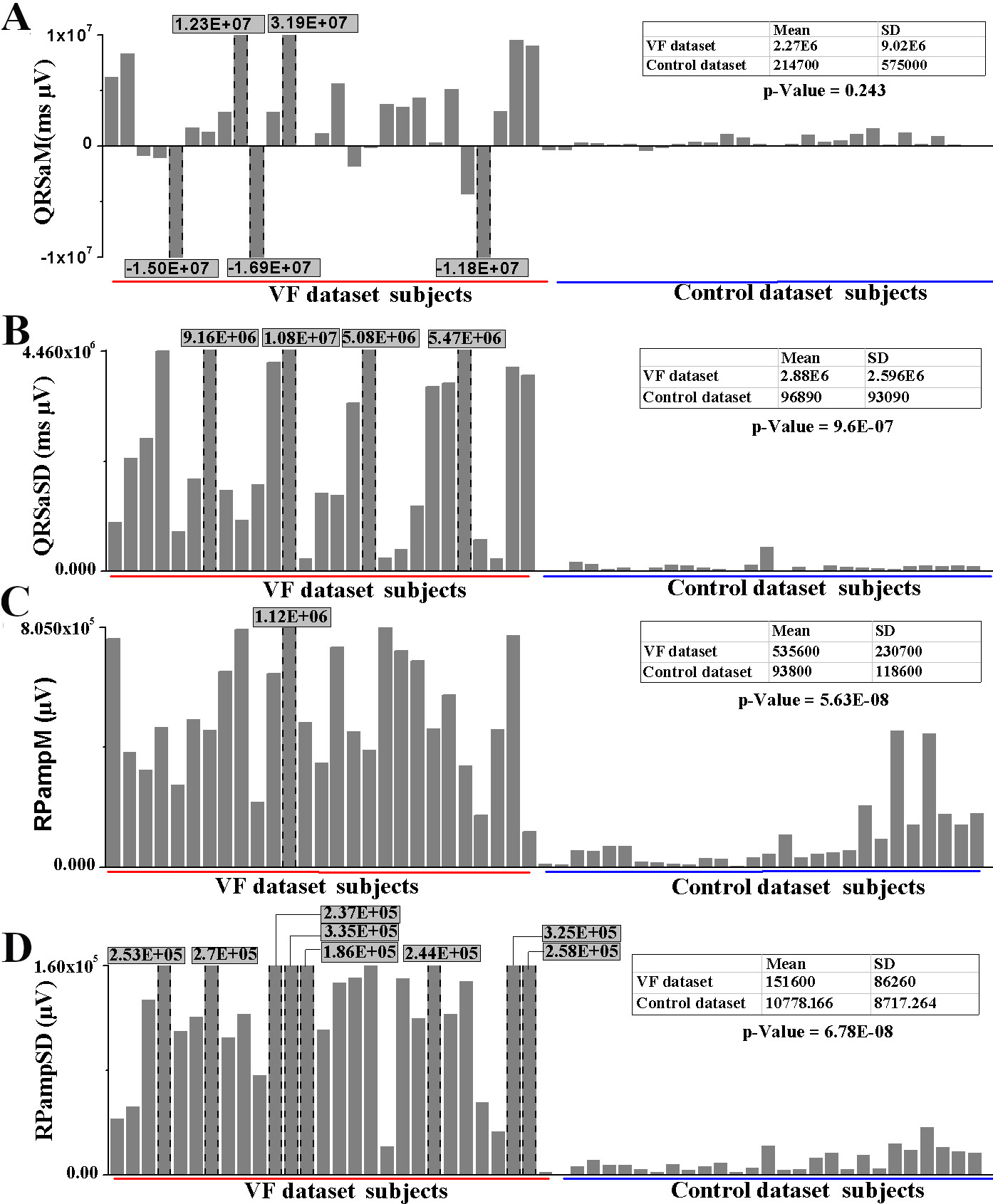
**

Supplementary Figure 4. (A) Mean of the QRS complex signed area (QRSaM). (B) Standard deviation of the QRS complex signed area (QRSaSD). (C) Mean of the R-peak amplitude (RPampM). (D) Standard deviation of the R-peak amplitude (RPampSD).

**ROC AUCs for SVM, kNN, RF, and NB.**

**
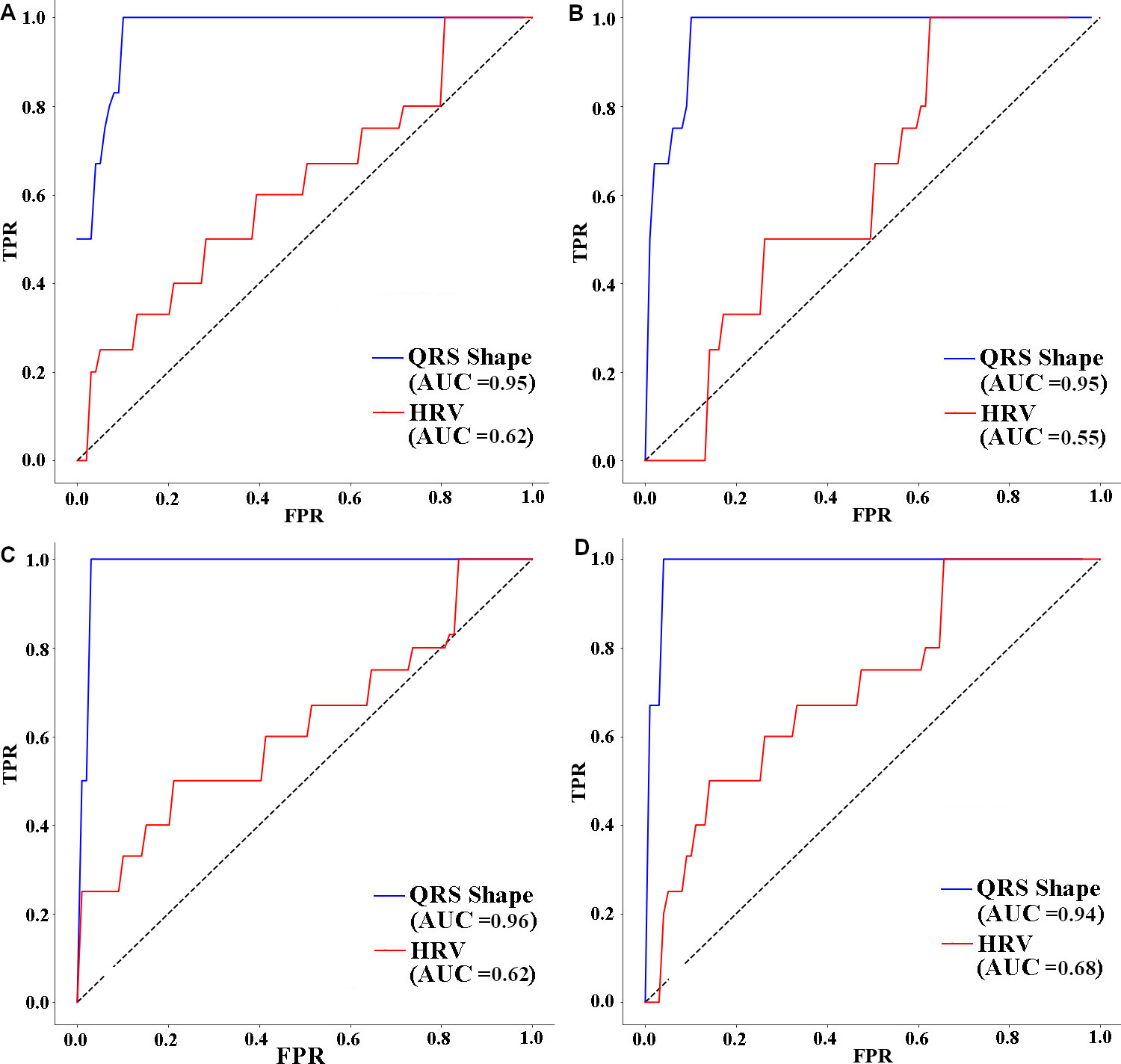
**

Supplementary Figure 5. Receiver operating characteristic area under curves (ROC AUCs) of (A) support vector machine (SVM), (B) k-nearest neighbors (kNN), (C) random forest (RF), and (D) Gaussian Naïve Bayes (NB). TPR – True Positive Rate and FPR – False Positive Rate.

# Supplementary Tables

The performance results for support vector machine (SVM), k-nearest neighbors (kNN), random forest (RF), and Gaussian Naïve Bayes (NB) using HRV and QRS shape features are provided below in supplementary Table 1 and 2, respectively. The average computational time for both fitting and prediction are also presented in ms.

Supplementary Table 1. The prediction results for the machine learning algorithms in predicting VF 30 s before its occurrence using HRV features, and their average execution time for fitting and prediction.

| **Algorithms** | **Sensitivity (%)** | **Specificity (%)** | **Accuracy (%)** | **AUC** | **Average time (ms)** | |
| --- | --- | --- | --- | --- | --- | --- |
|  |  |  |  |  | **fitting(training)** | **Prediction(testing)** |
| **ANN** | 65.68 | 98.44 | 72 ± 18.2 | 0.71 | 1545.04 | 0.72 |
| **SVM** | 61.92 | 74.53 | 63.6 ± 18.6 | 0.62 | 0.3 | 0.1 |
| **kNN** | 59.52 | 53.38 | 55.5±21.04 | 0.55 | 0.4 | 0.2 |
| **NB** | 63.32 | 98.33 | 71.04±18.13 | 0.62 | 0.4 | 0.09 |
| **RF** | 70.1 | 81.38 | 73±16.75 | 0.68 | 7.7 | 0.7 |

Supplementary Table 2. The prediction results for the machine learning algorithms in predicting VF 30 s before its occurrence using QRS shape features, and their average execution time for fitting and prediction.

| **Algorithms** | **Sensitivity (%)** | **Specificity (%)** | **Accuracy (%)** | **AUC** | **Average time (ms)** | |
| --- | --- | --- | --- | --- | --- | --- |
|  |  |  |  |  | **fitting(training)** | **prediction(testing)** |
| **ANN** | 98.4 | 99.04 | 98.6 ± 4.7 | 0.99 | 1505.1 | 0.7 |
| **SVM** | 96.49 | 99.49 | 97.6 ± 6.5 | 0.95 | 0.2 | 0.1 |
| **KNN** | 96.14 | 93.13 | 94.7 ± 8.9 | 0.95 | 0.3 | 0.2 |
| **NB** | 100 | 91.71 | 94.9 ± 8.6 | 0.96 | 0.4 | 0.09 |
| **RF** | 97.92 | 93.2 | 95.7 ± 8.7 | 0.94 | 7.3 | 0.6 |
